# The use of embodied self-rotation for visual and spatial perspective-taking

**DOI:** 10.3389/fnhum.2013.00698

**Published:** 2013-11-05

**Authors:** Andrew Surtees, Ian Apperly, Dana Samson

**Affiliations:** ^1^Faculté de Psychologie et des Sciences de l’éducation, Institut de Recherche en Sciences Psychologiques, University Catholique de LouvainLouvain-la-Neuve, Belgium; ^2^School of Psychology, University of BirminghamBirmingham, UK

**Keywords:** visual perspective-taking, spatial perspective-taking, embodied self rotation, theory of mind, level-2 perspective-taking, perspective-taking

## Abstract

Previous research has shown that calculating if something is to someone’s left or right involves a simulative process recruiting representations of our own body in imagining ourselves in the position of the other person (Kessler and Rutherford, 2010). We compared left and right judgements from another’s spatial position (spatial perspective judgements) to judgements of how a numeral appeared from another’s point of view (visual perspective judgements). Experiment 1 confirmed that these visual and spatial perspective judgements involved a process of rotation as they became more difficult with angular disparity between the self and other. There was evidence of some difference between the two, but both showed a linear pattern. Experiment 2 went a step further in showing that these judgements used embodied self rotations, as their difficulty was also dependent on the current position of the self within the world. This effect was significantly stronger in spatial perspective-taking, but was present in both cases. We conclude that embodied self-rotations, through which we actively imagine ourselves assuming someone else’s position in the world can subserve not only reasoning about where objects are in relation to someone else but *also* how the objects in their environment appear to them.

## INTRODUCTION

Human beings operate in complex social and spatial environments. In order to be successful, we must navigate our way around this complex world, in which other people are particularly important. Cooperation and competition are thought to have played a vital role in our evolution (Tomasello, 2008). In order to cooperate with and compete against others we often need to represent their perspectives. A minimal definition of a perspective is that it is someone’s relationship with objects and/or other people in their environment (Surtees et al., 2013). A perspective can be related to the visual experiences of an individual; famously in developmental psychology, Piaget and Inhelder (1956) asked children to judge how the experimenter *saw *an array of three mountains. Equally, a perspective can be related to the spatial location of an object; work on frames of reference has focused on people’s sensitivity to whether an object is located above or below, or to the left or the right of someone (Carlson-Radvansky and Jiang, 1998; Levinson, 1996). It is clear that a mature system for visual perspective-taking at times necessitates processing beyond the spatial relations between a person and the objects within their environment. Take for example a woman who hands her elderly husband his glasses to examine a passage in a book that, while it looks perfectly clear to her, she knows will appear blurry to him. In contrast to these special cases, however, there are a multitude of everyday social situations where rapid decision-making about approximations to other people’s visual experiences can be made simply on the basis of spatial relations and orientations. In this paper, we build on recent work comparing visual and spatial perspective-taking judgements (Kessler and Thomson, 2010; Kessler and Rutherford, 2010; Michelon and Zacks, 2006; Surtees et al., 2013) and examine the role for embodiment and rotation in visual and spatial perspective judgements.

### VISUAL PERSPECTIVE-TAKING

Since Piaget’s early description of children as egocentric (Piaget and Inhelder, 1956), a lot of focus has been placed on the age at which children first begin to understand that others do not share their own view (Flavell et al., 1981) or a good view (Light and Nix, 1983) of the world. Such judgements are thought to require children to have a Theory of Mind (Hamilton et al., 2009), that is to understand that other people are independent actors and that their behavior is dependent upon their own mental states (Premack and Woodruff, 1978) as well as the particular, current, state of the world. Whilst much of the focus in the literature on Theory of Mind has been on children’s ability to reason about beliefs in general, and False Beliefs in particular (Wimmer and Perner, 1983), successful reasoning based on the visual perceptions of others similarly requires us to be sensitive to their mental states and to overcome our own, egocentric biases (Surtees and Apperly, 2012).

Research with children and non-human animals suggests that perspective-taking is not a unitary ability (Masangkay et al., 1974; Flavell, 2000; Call and Tomasello, 2008). Flavell and colleagues (Masangkay et al., 1974; Flavell et al., 1981) make a distinction between level-1 and level-2 perspective-taking. Level-1 perspective-taking requires understanding of *what *can be seen, simply knowing which objects in the world are visually accessible to another person. Masangkay et al. (1974) showed children as young as 3 to be able to successfully report that an adult could see a dog pictured on the reverse of a card when they themselves saw a cat on its obverse. Children of this age were, however, unable to report that a picture of a turtle on a flat-lying card would look upside down to the adult when it looked the right way up to them. This latter task reflects level-2 perspective-taking, judging *how* someone sees the world, specifically judging that a single object can be represented differently by two different people based on their viewpoint in the world. The emergence of level-2 perspective-taking has been associated with other Theory of Mind developments that also occur around the age of four (Perner, 1991), such as False Belief reasoning (Wimmer and Perner, 1983), reasoning about the difference between appearance and reality (Flavell et al., 1983). Similarly, a number of other cognitive abilities significantly progress at this age, such as counterfactual thinking (Riggs et al., 1998), early reasoning about regret (Weisberg and Beck, 2010) and also executive functioning (Espy, 1997; Kirkham et al., 2003). The distinction between level-1 and level-2 perspective-taking appears not to be merely linked to children’s development, with many non-human animals, such as chimpanzees (Tomasello et al., 2003), goats (Kaminski et al., 2001), dogs (Hare and Tomasello, 2005) and Western Scrub-Jays (Emery and Clayton, 2004) showing level-1, but as yet no evidence of level-2 abilities. Similarly, infants (Song and Baillargeon, 2008) and adults (Samson et al., 2010) seem to be spontaneously sensitive to whether or not someone sees a given object, but again there is no such evidence for level-2 perspective-taking (Surtees et al., 2012a). It is this convergence of evidence that has led Apperly and Butterfill (2009) to suggest that the distinction between level-1 and level-2 perspective-taking may demarcate a signature limit on efficient theory of mind, such that level-2 judgements are always demanding of cognitive resources. In the current paper, we examine level-2 type judgements. Specifically judgements of how a numeral looks to someone else.

### SPATIAL PERSPECTIVE-TAKING

For Spatial perspective-taking here we mean the ability to understand the spatial relationship between an individual and the objects in their environment (sometimes spatial perspective-taking is used to refer to mentally occupying another’s position in space; Kessler and Wang, 2012). Unlike for visual perspectives, the content of Spatial perspectives is non-mental. A Spatial perspective is solely and definitively prescribed by the exact spatial relationship between a person and objects around them, rather than what they think about those objects. Whilst we may use our understanding of how others perceive the world around them to inform our judgements of where items are located in space relative to them, it is not necessary to do so. A book may remain to the front and the left of someone, regardless of whether they have perfect vision, suffer from short-sightedness or are blind. Similarly, if someone were to close their eyes, we should understand that they no longer have a visual perspective on the world, but maintain their spatial perspective. For this reason, spatial perspectives are not necessarily linked to individual people (Surtees et al., 2012b). A book can be located to the front and left of a chair in the very way in which it can be located to the front and the left of a person. Consequently, spatial perspectives have been most commonly considered in terms of frames of reference. A frame of reference is a set of axes upon which to consider the location of objects (Levinson, 1996, 2003). These axes can be absolute, defined by an unchanging element of the environment- Birmingham is located to the North of Brussels, regardless of where we are. They can be relative, defined by the position of objects in relation to the viewer- you cannot see the Manneken Pis if you stand on the Grand Place in Brussels because the Hotel de Ville is in front of it. Or they can be intrinsic, defined by one of the objects we are reasoning about- I can move the Palais Royal from being behind me to being in front of me by the simple expedient of turning myself around. It is these *intrinsic *frames of reference that incur spatial perspectives. Calculating an intrinsic reference frame requires understanding the relationship between an individual person or object and their environment. Spatial frames of reference are calculated automatically following the use of prepositions (Carlson-Radvansky and Jiang, 1998), requiring inhibition to choose the most appropriate frame. Both adults (Carlson-Radvansky and Irwin, 1993) and children (Surtees et al., 2012b) are known to be concurrently sensitive to multiple frames of reference. Like for visual perspective-taking, there is evidence that children do not necessarily use all aspects of a frame of reference at the same age (Hands, 1972; Harris and Strommen, 1972; Cox, 1981; Bialystok and Codd, 1987). They show a preference for the intrinsic frame of reference in early childhood and also learn the spatial referents “in front” and “behind” (Harris and Strommen, 1972; Cox, 1981; Bialystok and Codd, 1987), before the referents “left of” and “right of” (Hands, 1972). Interestingly, adults seem spontaneously sensitive to other people’s spatial perspectives. Tversky and Hard (2009) found that adults described objects as being to the left or right of a person even though the task only asked them to describe the location of an object.

### PROCESSES FOR VISUAL AND SPATIAL PERSPECTIVE-TAKING

Whilst much of the focus in the visual perspective-taking literature has been on the conceptual demands of understanding other people’s minds, it is clearly of importance to understand the cognitive architecture that allows us to represent others’ point of view (Kessler et al., under review). Such processing must take into account complex relationships between individuals and objects within their spatial environment. Recently, a number of studies have looked to identify the different processes for visuo-spatial perspective-taking. Michelon and Zacks (2006) proposed 2 kinds of visuo-spatial perspective-taking processes. The first of these, equivalent to level-1 visual perspective-taking, was used when adults had to judge if an object could be seen or not. This process was sensitive only to the distance between the target other and the object about which the perspective was taken. It was concluded that this process involved tracing the line of sight of the avatar. A second process was sensitive to the angular disparity between the participant and the other person in the scene and was used when participants had to judge if a specified object was to the left or right from the avatar’s position. This second process was concluded to require mental self rotation to align one’s own perspective with that of another. Whilst the exact question was if the other saw the object as on its left or right, it is clear that this second judgment is primarily spatial in nature and equivalent to a purely spatial judgment of whether the object was to the other’s left or right. Michelon and Zacks’s (2006) findings are in line with previous evidence of the effect of angular disparity on spatial judgements (Huttenlocher and Presson, 1973; Kozhevnikov and Hegarty, 2001; Keehner et al., 2006) and the identification of both visuo-spatial perspective-taking processes has since been replicated by Kessler and colleagues (Kessler and Rutherford, 2010; Kessler and Thomson, 2010; Kessler and Wang, 2012). In a recent study, we (Surtees et al., 2013) looked to further delineate these processes, and in particular examined whether the differences found by Michelon and Zacks (2006), Kessler and Thomson (2010) were primarily caused by judgements being of a visual vs. spatial nature, or whether they were primarily caused by judgements being of an early developing kind or a later developing kind. We found that spatial perspective judgements of an object as being in front of, or behind, like visual perspective judgements of whether something was visible, were not dependent on the angular disparity between the self and other. The difficulty of visual judgements of how a numeral appeared, on the other hand, like spatial judgements of something as being to the left or to the right for someone, were dependent on this angular disparity. We concluded that the selection of processing strategy was not determined by the nature of the content, as mental or non-mental, but rather by the specific task requirements and the degree to which simple features could be used. A rotational mechanism seemed to be the default method for only two kinds of judgements; level-2 visual perspective judgements of *how *something appeared to someone else and spatial judgements on the left to right dimension of an intrinsic frame of reference.

### EMBODIED SELF ROTATION VS. VIEWPOINT ROTATION

Difficulty based on angular disparity could be indicative of three different types of rotational strategies. The first is a mental self rotation, which uses an embodied representation of the self that is then rotated to the current bodily position of the target perspective (Kessler et al., under review). Such a process uses motor representations to imagine transporting ourselves to another’s position (Kessler and Thomson, 2010) and then simulates a self perspective from that new position. The second is a mental object rotation, through which we rotate the world from the angle of the target perspective to our own current position (Kessler et al., under review). Finally, the third is a mental viewpoint rotation (Kessler et al., under review), through which we use visuo-spatial cues to calculate a viewpoint in a given position without occupying that point of view in an embodied way. Only the first of the three strategies would require embodied self representations. Kessler and Thomson (2010) and Kessler and Rutherford (2010) used an innovative method to investigate whether mental self rotation was used for left and right judgements. Varying the angle of participants’ own bodies in relation to the screen, whilst keeping head position fixed, they reasoned, would only affect performance if mental self rotation was employed. They found (Kessler and Rutherford, 2010) that even though the visual impression remained the same (because the head position was fixed) across conditions, participants’ performance varied as a function of their own body angle, with better performance when their own body posture more closely matched that of the avatar. They concluded from this that judging if an object was to the left or the right of someone else involves an embodied process of self rotation to align our perspective with theirs. They found no impact of their body rotation manipulation on judgements of whether an object was visible to the avatar or not. This is perhaps not surprising as these judgements are not affected by angle at all.

### THE CURRENT STUDY

The aim of the current study was to test whether embodiment is also used in visual perspective-taking. In the current study, we adapted Kessler and colleagues’ (Kessler and Rutherford, 2010; Kessler and Thomson, 2010) body posture manipulation to compare its effect on two kinds of perspective-taking task. When participants judged if an object was to an avatar’s left or right (spatial perspective judgement), we predicted that they would use embodied self rotation. We expected that their performance would be affected not only by the angle of disparity between the avatar’s position and participants’ own position, but also by participants’ own body posture- with better performance when their own body posture was more similar to that of the avatar (as found by Kessler and Rutherford, 2010; Kessler and Thomson, 2010). When participants judged how a number looked to the avatar (visual perspective judgement), we predicted that judgements would again be affected by angular disparity (as found in Surtees et al., 2013). Our central research question was whether this process was embodied or not. If these judgements were also affected by body posture, it would indicate a common embodied self rotation process implicated in both visual and spatial perspective-taking. If these judgements were independent of body posture consistency, this would suggest that these judgements involved non-embodied viewpoint rotation.

## EXPERIMENT 1

### MATERIALS AND METHODS

#### Participants

Participants were 40 undergraduate students (11 male) from the University catholique de Louvain, Belgium. They all participated in the study in exchange of course credit or a small honorarium of 8 Euros. Participants had an average age of 20.77 years (range 18–25). One participant was not included in the final sample on the basis of performing below chance.

#### Stimuli

**FIGURE 1:**
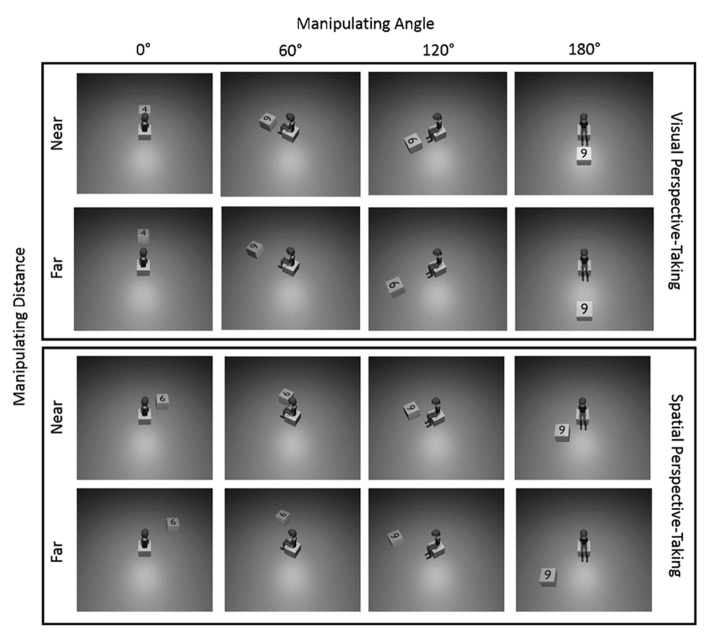
**Example stimuli from Experiment 1.** Here all examples show anticlockwise rotation. An equal number of stimuli showed clockwise rotations. The Visual perspective-taking condition also included stimuli in which the block/number was located behind the avatar.

In all of the pictures that participants saw, an avatar was placed in the center of a featureless room (see **Figure 1**). The stimuli were created using Blender (). The room also contained a single cube, with a numeral written on its top-most face (4, 6, 7 or 9). Within each stimulus, we varied two features orthogonally. Angular disparity between the participant and the avatar was varied through the positioning of the virtual camera in relation to the avatar, creating angles of four different magnitudes: 0°, 60°, 120°, 180°. For angular disparity of both 60° and 120°, separate stimuli were created showing the avatar in clockwise and anticlockwise variants, crucial for evaluating our embodiment hypothesis. We also varied distance, by placing the block at one of two distances from the avatar: “Near” and “Far,” where the Far condition was placed at a distance that was twice as far within the virtual world as the Near condition. In the spatial condition, an equal number of stimuli placed the block/number to the left and to the right of the avatar, at an angle of 45° from the avatar and always in front of him. In the visual condition, stimuli showed the block/number to be directly in front, or directly behind the avatar.

**FIGURE 2:**
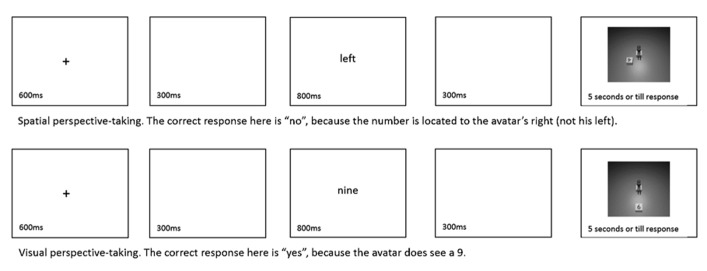
**Basic procedure in Experiment 1.** Participants verified whether a cue they saw matched a picture that followed. Note, on every trial, before these slides, participants were cued to the rotation they had to make.

**FIGURE 3:**
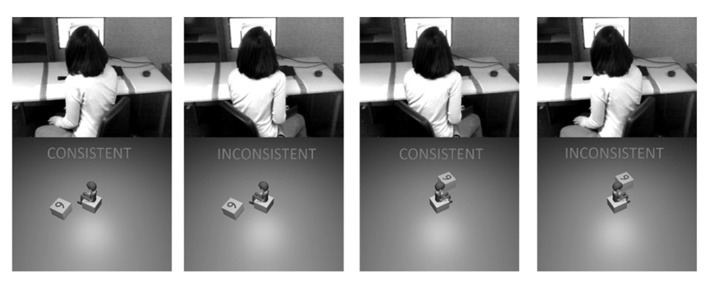
**Demonstration of Consistency effect.** By turning anticlockwise, pictures showing the avatar also rotated anticlockwise are Consistent and those where the avatar has turned anticlockwise are Inconsistent. A clockwise turn has the opposite effect. Note how the specific picture stimulus, the direction of the turn and the visual impression for the participant are independent of Consistency.

Participants were randomly divided into two groups, Visual or Spatial. Before the experiment, all participants were given the same basic information, that they would be performing a perspective-taking task. Participants were sat in a rotating chair, with a red rectangle attached to the floor at approximately 60° angle to their right and a blue rectangle at approximately 60° to their left. They placed their chin on a chin-rest (located 50 cm from the screen) on every trial. After further instruction, giving example procedures and the correct answer, all participants completed 16 practice trials without rotation, 8 practice trials with rotation and finally 256 experimental trials divided into four blocks. All trials followed the same basic procedure (see **Figure 2**). Participants were first of all cued with a picture showing a red or blue square with a schematic illustration of a person (adapted from Kessler and Thomson, 2010). Participants had been instructed that the red picture meant they should rotate their body to the left/anticlockwise and place their feet on the red rectangle on the floor, they were instructed to keep the mouse on their lap (see **Figure 3**). The blue picture conveyed the same instruction, but to the right/clockwise. These rotations meant that participants’ own body orientation varied from approximately 60° clockwise to approximately 60° anticlockwise in relation to the screen for every trial. Importantly, though, by keeping their chins on the chin rest, participants’ visual impression did not change (beyond the variations in the stimuli type presented on the screen). Following the rotation cue, participants saw a further screen, asking whether they had made the rotation, this required a mouse click to progress. The experimenter observed a sample of these rotations and saw no cases in which participants made errors in their rotations (this included at least 20 consecutive trials for each participant). Following this stimulus, the standard trial sequence (Surtees et al., 2013) was presented (**Figure 2**). A fixation cross was followed by a cue (for spatial, left or right; for visual, four, six, seven, or nine). This cue was followed by the picture itself. In response to the picture, participants pressed the left mouse key to indicate that it matched the cue and the right mouse key to indicate that it did not. Participants received feedback during practice, but not during the experiment itself.

For the Spatial condition, trials were equally and orthogonally divided on four experimental factors. There were an equal number of trials in which the cue did and did not match the picture (Match/Mismatch). An equal number of trials of each of the Angles (0°, 60°, 120°, 180°), of which the angles 60° and 120° were equally often clockwise or anticlockwise rotations. An equal number of stimuli showed each Distance (Near, Far). Finally, an equal number of trials required a left (red) or a right (blue) rotation. Note, that our variable of particular interest- the consistency between avatar and participant rotation- was varied through a combination of Angle and Rotation. Consistent trials occur with a left rotation of the participant and an anticlockwise rotation of the avatar *or* with a right rotation of the participant and a clockwise rotation of the avatar. This means that the factor Consistency was independent of stimulus and independent of participant rotation. Stimuli were also varied on whether the cue/object was left or right and whether the number was a 4, 6, 7 or 9.

For the Visual condition, trials were again equally divided between Match and Mismatch trials. For Match trials, stimuli were varied exactly as above, save for the fact that the block/number was always directly in front of the avatar. For half of mismatch trials, “number mismatch trials,” the same stimuli were presented, with the block/number directly in front of the avatar, but the preceding cue being a different number to that seen by the avatar in the picture. In the other half, “location mismatch trials,” the cue would have been correct had the avatar been looking at the number, however, it was placed directly behind him. This manipulation meant that participants had to take into account the avatar’s view and could not use the rotation of the number alone as a cue to the correct answer.

In summary, the Visual and the Spatial conditions for Match trials (those to be analyzed) were identical other than features necessary for the specific judgements. Both conditions included a range of angles from 0° to 180°, to confirm that rotation was being used for the task at hand. By manipulating the position of the participant in relation to the screen, and the positioning of the avatar on the screen, we varied consistency between body postures. This was independent of Angle, Distance, Rotation, Task Content (Visual, Spatial), Number, Cue and Direction, so that any influence could only be the result of the congruency between the embodied state of the participant and the avatar (see **Figure 3**).

## RESULTS

Only Match trials- those in which the cue matched the picture- were included in the final analysis. Outliers were excluded from the analysis of response times on the basis of being more than 2.5 standard deviations away from the mean response time (2.9% for visual, 2.7% for spatial), as were incorrect responses.

Our first analyses investigated the effects of Angle and Distance on perspective-taking. Particularly important here are the effects of Angle and any interaction between Angle and Content. A linear effect of Angle would be representative of participants using some form of rotation to complete the task. This analysis does not investigate the embodied nature of the process.

A 4 × 2 × 2 ANOVA with Response Time as a dependent variable, Angle (0°, 60°, 120°, 180°) and Distance (Near, Far) as within subjects factors and content (Visual, Spatial) as a between subjects factor revealed a main effect of distance, *F*(1, 38) = 10.46, *p *= 0.003, η*p*^2^**= 0.216, with shorter avatar-object distances processed more quickly^1^. There was also a main effect of Angle, *F*(3, 114) = 37.71, *p* < 0.001, η*p*^2^ = 0.498, which represented a linear trend. There was also a main effect of content, with Visual judgements being responded to more quickly, *F*(1, 38) = 12.89, *p* = 0.001, η*p*^2^ = 0.253.

**FIGURE 4:**
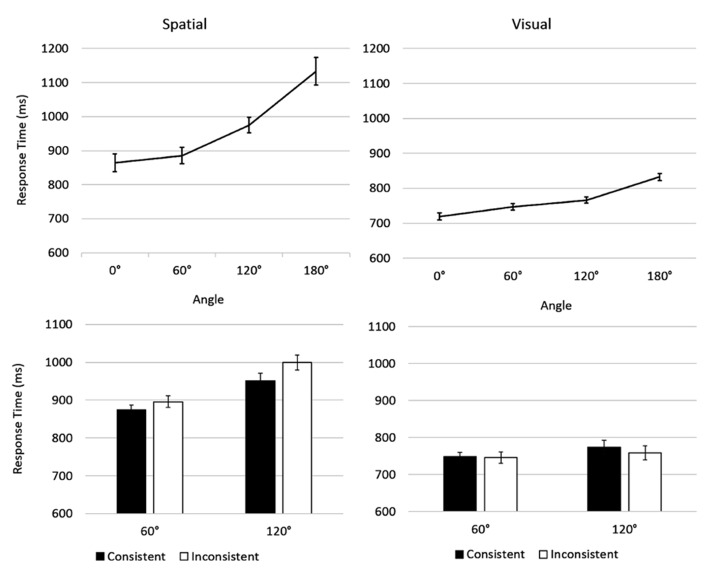
**Both visual (right) and spatial (left) perspective-taking showed a significant, linear effect of angle.** In spatial perspective judgements there was a trend for participants performing better if their own body posture was consistent with that of the avatar, in the visual condition, there was no such trend. Error bars show within-subjects standard error of the mean.

An interaction between Angle and Content, *F*(3, 114) = 7.43, *p* = 0.004, η*p*^2^ = 0.163, revealed a different relationship with Angle for each Content. For both Visual, *F*(1, 19) = 41.36,* p *< 0.001, and Spatial, *F*(1, 19) = 31.83,* p *< 0.001, perspective-taking the relationship with Angle fitted a linear trend. We investigated this relationship further, by computing separate *t*-tests for adjacent angles for each content. For Spatial perspective-taking, the strongest effect was for participants being slower at 120° than 60°, *t*(19) = 5.67, *p* < 0.001, with a less strong, but still significant effect of 180° being slower still *t*(19) = 5.67, *p* = 0.003. Though responses at 0° were the slowest, these were not significantly slower than at 60°, *t*(19) = 1.361, *p* = 0.190. Visual perspective judgements showed a different pattern of performance (see **Figure 4**). Here difference was greatest for judgements at 180° being slower than at 120°, *t*(19) = 4.75, *p* < 0.001. There was a trend for an effect of faster judgements at 60° than 0°, *t*(19) = 1.934, *p* = 0.068 and no significant effect between 60° and 120°, *t*(19) = 1.341, *p* = 0.196, though again the larger angle produced a numerically longer response time. The interaction between Distance and Content, *F*(1, 38) = 7.00, *p *= 0.012, η*p*^2^ = 0.156, illustrated that there was an effect of Distance on Visual, *F*(1, 19) = 20.85, *p *< 0.001, η*p*^2^ = 0.543, but not Spatial, *F*(1, 19) = 1.47, *p *= 0.705, η*p*^2^ = 0.008, judgements.

Error rates across conditions were generally low and did not contradict the findings from response time (see **Table 1**).

**Table 1:** ercentage error rates (standard deviations) from Experiments 1 and 2.

**Experiment 1**
	0°	60°	120°	180°	Consistent	Inconsistent
Spatial	5 (5)	3.59 (4)	4.84 (5)	7.34 (7)	4.34 (5)	4.06 (4)
Visual	1.87 (3)	3.75 (5)	1.41 (3)	3.28 (3)	2.03 (3)	3.13 (4)
**Experiment 2**
	**120° Consistent**	**120° Inconsistent**	**150° Consistent**	**150° Inconsistent**
Spatial	3.65 (4)	7.03 (9)	2.60 (3)	5.99 (7)	
Visual	3.65 (5)	5.99 (5)	3.13 (3)	3.65 (6)		

Trials in which angular disparity was either 60° or 120° could be either Consistent or Inconsistent on the basis of whether participants have rotated their body to the left or to the right. Analysing this subset of trials with Consistency as an additional factor can test the role of embodiment. A 2 × 2 × 2 ANOVA was completed with Content as a between subjects factor and Consistency (Consistent, Inconsistent) and Angle (60°, 120°) as within subjects factors. A main effect of Angle, *F*(1, 38) = 25.55, *p* < 0.001, η*p*^2^ = 0.402, was moderated by an interaction between Angle and Content, *F*(1, 38) = 10.63, *p* = 0.002, η*p*^2^ = 0.219. Over this smaller range of angles, the effect was only significant in the Spatial domain, *F*(1, 19) = 33.07, *p* < 0.001, η*p*^2^ = 0.635, not the Visual domain, *F*(3, 19) = 1.69, *p* = 0.210, η*p*^2^ = 0.081. There was no significant effect of Consistency, *F*(1, 38) = 1.30, *p* = 0.261, η*p*^2^ = 0.033, but there was a trend for an interaction between Consistency and Content, *F*(1, 38) = 3.63, *p* = 0.064, η*p*^2^ = 0.087. This illustrated a trend for Consistent trials being easier than Inconsistent, but only in the Spatial condition, *F*(1, 19) = 3.041, *p* = 0.097, η*p*^2^ = 0.138 (see **Figure 4**).

In Experiment 1, Visual perspective-taking, but not spatial perspective-taking showed an effect of distance. This was surprising and had not been evidenced in our previous study (Surtees et al., 2013), in which we found a significant effect of distance that did not differ across conditions. One possibility is that having fewer conditions here (two rather than four) has given a greater power to identify a difference. This is supported by the fact that in the spatial condition of Surtees et al. (2013), at two angles (0° and 120°), judgements at shorter distances were actually more difficult than at longer distances. This was never the case in the visual condition, where further distance always conferred greater difficulty. Both Visual and Spatial perspective-taking showed a strong and linear effect of angular disparity between the participant and the avatar on the screen in front of them, replicating the findings of Surtees et al. (2013) and suggesting a rotational process was employed. That is not to say, however, that this relationship was identical. For Spatial perspective-taking, the strongest effect was between the two mid-range angles, 60° and 120°. For Visual perspective-taking, this difference was not significant, instead it was the difference between 120° and 180° that was most strongly significant. This is in some ways surprising, as this difference was not found by Surtees et al. (2013) who used the very same stimuli. One possibility is that the physical rotations (regardless of direction) had a different effect on the rotational processes of Visual and Spatial perspective. Specifying exactly how is very speculative at this stage, but one possibility is that for Spatial perspective taking, the 60° condition was made artificially easy because here the character’s basic body posture matched the participant’s.

Experiment 1 also showed a trend for an interaction between Consistency and Content suggesting that visual and spatial perspective-taking may be embodied to a different degree. Spatial perspective-taking showed a trend for an effect of Consistency. Participants trended toward performing better when their own position was aligned with that of the avatar. From this, we tentatively concluded that spatial perspective-taking recruited an embodied self rotation process, while visual perspective-taking recruited a (non-embodied) viewpoint rotation process. However, as the trends were non-significant, it also remains possible that our test was insensitive to differing embodied effects (for we should expect an effect of body posture consistency at least in the spatial condition to replicate the findings of Kessler and Rutherford, 2010; Kessler and Thomson, 2010). Also, our first experiment investigates one specific circumstance when we have to confirm a pre-defined proposition for the other’s perspective (our task required a verification, yes/no, judgement). It is possible that actively calculating another person’s perspective uses embodiment to a different degree. In Experiment 2, we addressed both the concern over lack of sensitivity and over perspective confirmation vs. calculation by using a forced choice methodology. As well as removing the verification aspect of the procedure, this method has the advantage of increasing the power (as all responses are permissible in the final analysis). Also to increase power, we removed the stimuli showing the avatar at 0° and 180° (as these only tested for rotation, not embodiment *per se*) and tested only at angles higher than 90°, those which previous studies have found to show clearest embodiment effects (Kessler and Thomson, 2010; Kessler and Rutherford, 2010).

## EXPERIMENT 2

### MATERIALS AND METHODS

#### Participants

Participants were 32 undergraduate students (9 male) from the University catholique de Louvain, Belgium. They all participated in the study in exchange for a small honorarium of 8 Euros. Participants had an average age of 21.93 years (range 18–26).

#### Design and procedure

The design of Experiment 2 was identical to that of Experiment 1, other than the following details. Instead of making responses to a preceding cue, here participants made a forced choice response. For Spatial perspective-taking, this meant pressing the left button on the mouse when the number was located to the left of the avatar and the right when it was to his right (note here that effects of spatial compatibility, Simon, 1969, were controlled across body posture consistency). For Visual perspective-taking, it meant pressing the left button when the number the avatar saw was a number six and the right when he saw a number nine. In this case, only stimuli where the avatar saw a six or nine were included and they were always placed in front of him (and displaced to the left or right in the spatial condition). After completing 24 practice trials (as in Experiment 1, 16 with the task alone, 8 with rotation), participants completed 96 experiment trials. Fewer trials were needed here as all trials were included in the final analysis (the analysis of Consistency has more power). New stimuli were created that had the avatar placed at either 120° or 150° angle from the participant. Again, participants were cued before each trial to rotate to the left or right, again placing their feet on the mat at an angle of approximately 60° to the screen.

## RESULTS

Again, trials in which participants made incorrect responses were excluded (see **Table 1**), as were trials in which response time was more than 2.5 standard deviations away from the mean (3.2% for visual, 3.1% for spatial).

**FIGURE 5:**
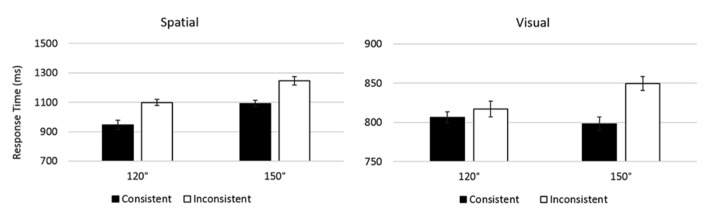
**Results of Experiment 2.** Error bars show within-subjects standard error of the mean. The effect of Consistency is greater for Spatial perspective-taking. There was no interaction between Angle and Consistency.

A 2 × 2 × 2 ANOVA with Content (Visual, Spatial) as a between subjects factor, and Angle (120°, 150°) and Consistency (Consistent, Inconsistent) as within subjects factors revealed an effect of Angle, *F*(1, 30) = 19.88, *p* < 0.001, η*p*^2^ = 0.399, 120° < 150° (**Figure 5**). This effect was moderated by an interaction with Content, *F*(1, 30) = 14.20, *p* = 0.001, η*p*^2^ = 0.321, showing the effect of Angle *was *significant for Spatial judgements, *F*(1, 15) = 18.95, *p* = 0.001, η*p*^2^ = 0.558, but not for Visual judgements, *F*(1, 15) = 1.11, *p* = 0.308, η*p*^2^ = 0.069. There was no interaction between Angle and Consistency, or Angle, Consistency and Content, *Fs* < 1.10, *ps* > 0.307, η*p*^2^ < 0.035.

Crucially, there *was* an effect of Consistency, *F*(1, 30) = 25.84, *p* < 0.001, η*p*^2^ = 0.463. This was moderated by a significant interaction, with Content, *F*(1, 30) = 11.42, *p* = 0.002, η*p*^2^ = 0.276. Investigating this interaction showed that while the size of this effect was numerically greater in the spatial condition, *F*(1, 15) = 18.79, *p* = 0.001, η*p*^2^ = 0.566, it was also significant in the Visual condition, *F*(1, 15) = 15.31, *p* = 0.001, η*p*^2^ = 0.505. As in Experiment 1, there was also an effect of Content, *F*(1, 30) = 9.79, *p* = 0.004, η*p*^2^ = 0.246, such that Visual perspectives were processed more quickly.

## DISCUSSION

Across two experiments, we investigated the degree to which perspective-taking required mental rotation and the degree to which that rotation was embodied. We tested this for two very different kinds of perspective-taking. We found further evidence that an explicitly spatial task recruited mental rotation. When participants judged whether an object was to the left or the right of an avatar it became increasingly more difficult as the angle of the avatar’s body became increasingly more different from the participant’s position. In addition to this, we found evidence that this rotational process was an embodied self rotation, as has previously been shown by Kessler and Thomson (2010), and Kessler and Rutherford (2010). Participants found it easier (statistical trend in Experiment 1 and significant effect in Experiment 2) to make spatial judgements when their own body posture more closely matched that of the avatar- even though this was manipulated independently of the visual impression of the scene. When participants completed a visual perspective-taking task, we also found evidence of rotation. Experiment 1 showed that it is harder to judge how a number appeared to an avatar whose angular viewpoint differed from one’s own to a greater degree. Perhaps most surprisingly, in Experiment 2, we showed that this process could also involve an embodied self rotation. In sum, findings for spatial perspective-taking suggested consistent use of embodied mental self rotation. For visual perspective-taking we evidenced the same process, but the strength of the effect was neither as strong nor as consistent as for spatial perspective-taking. The embodiment of this process was only evidenced in Experiment 2 and even here was not as strongly significant as for spatial perspective-taking.

### VISUAL PERSPECTIVE-TAKING

Judging how a numeral looks to someone else who does not view it from the same angle as us is a clear example of level-2 visual perspective-taking, knowing that a single object can make a different visual impression on two people who view it from different angles (Flavell et al., 1981). This process is known to be difficult both for children (Masangkay et al., 1974) and for adults (Surtees et al., 2012a). Developmentalists have tended to focus on the conceptual difficulties posed by holding two conflicting relationships on a single object (Perner, 1991) or on the demands in inhibiting a salient self perspective (Surtees et al., 2012a). Here we present evidence that one source of difficulty in these tasks is rotation. Whilst it is clear that in Flavell’s classic “turtle” task we have to understand that another person can represent the same turtle differently *and *inhibit a salient self-view of a turtle happily upstanding or disarmingly prostrate, we also need to mentally align how we see the world with how it is seen by the person with whom we are interacting. We replicate findings from our previous study (Surtees et al., 2013) that level-2 visual perspective judgements become more difficult as our angle becomes more different from that of the person whose perspective we take. In Experiment 1, we showed a linear effect of Angle on speed of responses.

Kessler and Rutherford (2010), Kessler and Thomson (2010) showed that judging that one object was on the left or the right from someone else’s point of view was affected by the participant’s current body angle in the world. Here we show that the same applies to judgements of visual perspectives. In Experiment 2, participants’ own body angle affected their ability to judge if a number looked like a six or a nine to an avatar on the screen. This is the first finding showing that a judgment of a purely mental state can also require us to align our bodies with that of someone else in the world. This suggests that, at least in some cases, to think of how someone else sees the world requires us really “putting ourselves into their shoes.” The effect of body posture consistency was however only significant in Experiment 2 and not in Experiment 1. There are two possible explanations to account for these discrepant results. One possibility is that Experiment 1 simply was not sensitive enough to demonstrate this effect. A second possibility is that the difference reflects the employment of different processes determined by surface demands of the situation. Experiment 1, in which participants have to hold in mind a cue (e.g., “nine” meaning that they have to verify if the object looked like a 9 to the avatar), may promote a different strategy from Experiment 2 in which participants’ judgements are solely based on the picture stimulus (here participants have to decide whether the object looks like a “6” or a “9” to the avatar when presented with the picture). In Experiment 1, participants could have used the cue to create a mental image of an expected stimulus and then used a geometrical comparison between this and the final picture. This would result in the observed effect of angle and the absence of effect of body posture consistency. In Experiment 2, on the other hand, the effect of body posture consistency seems to rule out that such geometrical comparison was used consistently across trials and participants. It is also possible that some participants used conditional rules to calculate visual perspectives (e.g., If he faces toward me then he does not see the same number as me), but our significant findings, of angular disparity in Experiment 1 and embodiment in Experiment 2 suggest this was not widely applied. We propose that level-2 visual perspective-taking requires flexible processing (Apperly and Butterfill, 2009) and its challenges may be met in a number of ways (Kessler et al, under review), and may be dependent on the precise requirements of a problem and even individual differences (Kessler and Wang, 2012). Further studies may look to experimentally manipulate strategy use through systematically priming the use of conditional rules, geometrical comparison and embodied self rotation or through using a dual-task situation to occupy resources for language, imagined spatial manipulation or proprioception.

### SPATIAL PERSPECTIVE-TAKING

Evidence that we use spatial alignments of perspectives to calculate the perceptions of others suggests perspective-taking is reliant on an understanding of the relationships between people and objects in space. There are, of course, many judgements that explicitly require us to use such relationships, with no pre-text of mental state use whatsoever. When I ask a colleague to pass the coffee cup that’s to her left, I’m using my understanding of her intrinsic frame of reference- her spatial perspective. Interestingly, some cultures do not use these spatial perspectives for these kinds of judgements, preferring the absolute reference frame- pass the cup that is nearer to the river than you are (Levinson, 1996; Bowerman and Choi, 2003). We show, here and across two experiments, that these judgements that something is to someone’s left or right require embodied self rotation. Like judgements of how a numeral looks, they are sensitive to both the angular disparity between us and the person whose perspective we take *and *to the consistency of our current body position (replicating the findings of Kessler and Thomson, 2010; and Kessler and Rutherford, 2010). It seems that to judge that a coffee cup is to someone’s right involves us imagining that we are where they are and then judging if the coffee cup would be to our left our right. Quite noticeably, the effects of angular disparity and body posture consistency were stronger in the spatial than visual perspective judgment conditions, suggesting that the use of embodied self mental rotation was a strategy more widely used across trials and participants. One possibility is that this is the result of us using our own body representations as a cue to remember the locations of left and right (in England and in Belgium, for example, a common strategy is to remind school children that your “right is the one you write with”). An interesting further question addresses whether such judgements of spatial perspectives are principally for or exclusive to reasoning about human others. There is good evidence that human and non-human spatial transformation do not necessarily use the same cognitive (Zacks et al., 2000) or neural (Zacks and Michelon, 2005) processes. On the other hand, no studies have examined the processes used for locating objects relative to other people or other objects (rather they have focused on identifying the left or right arm of a person vs. the left or right side that a handle of a cup is on). Similarly, we may predict a role for strategy (Kessler and Wang, 2012) and for specific expertise- such as a tennis fan who can quickly judge a ball as being to Novak Djokovic’s forehand (right) side or Rafael Nadal’s forehand (left) side or a naval officer who can quickly conclude that a shoal of dolphins is to the port (left) of the HMS Ark Royal.

### COMPARING VISUAL AND SPATIAL PERSPECTIVE-TAKING

*Similarities.* On the basis of the findings of Experiment 2 in which body posture consistency effects were found on both types of perspective-taking judgements, we have concluded that both Spatial perspective judgements of an object as being to the left or right of someone and visual perspective judgements of how something looks to them recruit processes including embodied self rotation. Under tightly controlled experimental conditions, in which participants take another’s perspective on multiple occasions, both sets of judgment are sensitive to the angular disparity between the target other person and the self viewpoint *and *to the current orientation of the self body. We suggest that an important step for each problem is to imagine ourselves in the position of the other.

*Differences. *It is clear that further processing beyond an embodied rotation is required to solve these problems and that this processing necessarily differs for each task. Mature visual perspective-taking must take into account individual characteristics of their target: blindfolds, blurred vision or a lack of attention can significantly change how we judge another’s visual perspective in a way that is not required for spatial perspective-taking. These extra demands of visual perspective-taking may be in part responsible for the fact that our embodiment effect was less reliable for visual than for spatial perspective-taking. In Experiment 1, there was no evidence of an effect of consistency of body posture for visual perspective-taking and the effect in Experiment 2 was significantly stronger for spatial perspective-taking. We follow Kessler and Wang (2012) in promoting the idea that in these effortful perspective-taking tasks strategies may differ between individuals and on the basis of specific task demands. Our experiments suggest that variable strategy use was more prevalent for visual than for spatial perspective-taking. We also found evidence that spatial perspective-taking was substantially more difficult than visual perspective-taking in both experiments. We believe the most parsimonious explanation of this is that we use embodied self rotation and then simulate the perspective from that position. As judging objects as being to one’s own left or right is likely to be more difficult than judging how a number looks (a simple, automatized reading process) this would explain the overall difference.

Visual and spatial perspective-taking also differed in the nature of their relationship with Angle. We concluded that both processes required rotation, based on their linear relationship with Angle in Experiment 1. There was, however an interaction between Angle and Content. Following up this interaction showed that the precise pattern of added difficulty gained with increasing angle was not identical between the two kinds of perspective-taking. Most notably, while the difficulty of taking spatial perspectives grew *most* substantially between 60° and 120°, for visual perspective-taking, this comparison did not reach significance. Similarly, while Experiment 2 showed a robust effect of Angle in spatial perspective-taking, this was not the case in visual perspective-taking. These findings differ somewhat from the findings of Surtees et al. (2013), in which we used a similar method without the physical act of rotating the body, although importantly, both studies show a basic linear relationship between angular disparity and the difficulty of visual perspective-taking. We suggest that this rotation may have made some difference to both the exact nature of processing difficulty at different angles and to the variability in responses. Exactly explaining these specific differences may require further study, but the matter of key importance is that minor experimental changes affected spatial and visual perspective-taking differently, further suggesting that though they adopt similar processes, there are still clear differences in the instantiation of these processes.

### THE DEVELOPMENT OF VISUAL AND SPATIAL PERSPECTIVE-TAKING

Identification of similar strategies for spatial judgements of left/right and visual judgements of *how* something looks to someone else is consistent with the developmental profile of these abilities. The ability to make left/right judgements (Hands, 1972) develops after the ability to make front/back judgements (Harris and Strommen, 1972; Cox, 1981; Bialystok and Codd, 1987). Similarly, judgements of *how* something looks (level-2 visual perspective-taking) are achieved after judgements of whether or not someone can see something (Flavell et al., 1981; Moll and Tomasello, 2006; Moll and Meltzoff, 2011). Our current findings imply one possible explanation for this. That the most common and robust method for achieving both of these processes requires embodied mental self rotation, suggests that it may be difficulties with this embodied rotation, rather than with perspective-taking *per se* that is evidenced in developmental studies. There is much debate and conflicting evidence regarding children’s abilities in object rotation (Perrucci et al., 2008), even after the age they pass standard perspective-taking tasks. To our knowledge, however, there has been no systematic investigation of their abilities at mental self rotation.

That success on level-2 visual perspective-taking tasks may be dependent on embodied self-rotation allows for one of two broad alternative explanations. Firstly, children may have the basic conceptual apparatus to succeed in level-2 perspective-taking situations, even before they pass, but this conceptual knowledge may be obscured by lacking the domain general ability to imagine rotating their position in the world. This alternative is supported by findings of precocious performance on a level-2 type task employing color filters with 3-year olds (Moll and Meltzoff, 2011), rather than angular differences in perspective. Secondly, embodied self rotation may play a causal role in children learning the abstract, non-spatial notion of perspective. This idea, that increased processing flexibility may play a crucial role in children’s development of complex concepts, has been suggested by Russell (1996) in relation to children’s agency helping them to learn about the world. Investigation of the relative development of rotation and effortful perspective-taking should tell us whether rotation is necessary for learning perspective concepts, necessary for achieving perspective transformations in young children or co-opted once adults have developed a range of perspective-taking strategies and have substantial executive resources.

## CONCLUSION

When we interact in complex social environments we undertake complex visuo-spatial reasoning which may or may not involve thinking about the mental states of other people. Taxing judgements of how the world appears to someone else and what things are located to the left or the right of them seem to involve a comparable process of embodied self rotation. We imagine ourselves in the position of a target other. To do this we take as a starting point the current position of our own body as well as the visual input of a scene in front of us. Embodied perspective-taking processes are robust processes effective in generating visual perspectives of anyone whose basic perceptual apparatus is the same as ours and generating spatial perspectives of anyone who shares our basic anatomy. That is not to say that these processes are the same *in toto, *but rather that they share common processing features and strategy use. These processes are relatively costly and solve problems that are beyond the abilities of very young children. Further studies may look to consider what in these processes responds solely to target human others (as opposed to objects), how we deal with special cases in which other’s perceptual access is compromised and how experts overcome the costly nature of this perspective-taking process.

## Conflict of Interest Statement

The authors declare that the research was conducted in the absence of any commercial or financial relationships that could be construed as a potential conflict of interest.

## Footnotes

1 In both experiments, all statistics are Greenhouse-Geisser corrected to guard against violations of sphericity.

## References

[B1] Apperly I. A. Butterfill S. A. (2009). Do humans have two systems to track beliefs and belief-like states? *Psychol.Rev.* 116 953–970 10.1037/a0016923 19839692

[B2] Bialystok E. Codd J. (1987). Children’s interpretations of ambiguous spatial descriptions. *Br. J. Dev. Psychol.* 5 205–211 10.1111/j.2044-835X.1987.tb01055.x

[B3] Bowerman M. Choi S. (2003). “Space under construction: language-specific spatial categorization in first language acquisition,” in *Language in Mind* eds D. Gentner and S. Goldin-Meadow (Cambridge, MA: MIT Press) 387–428

[B4] Call J. Tomasello M. (2008). Does the chimpanzee have a theory of mind? 30 years later. *Trends Cogn. Sci.* 12 187–192 10.1016/j.tics.2008.02.010 18424224

[B5] Carlson-Radvansky L. A. Irwin D. E. (1993). Frames of reference in vision and language – where is above. *Cognition* 46 223–244 10.1016/0010-0277(93)90011-J 8462273

[B6] Carlson-Radvansky L. A. Jiang Y. H. (1998). Inhibition accompanies reference-frame selection. *Psychol. Sci.* 9 386–391 10.1111/1467-9280.00072

[B7] Cox M. V. (1981). Interpretation of the spatial prepositions in front of and behind. *Int. J. Behav. Dev.* 4 359–368 10.1177/016502548100400304

[B8] Emery N. J. Clayton N. S. (2004). The mentality of crows: convergent evolution of intelligence in corvids and apes. *Science* 306 1903–1907 10.1126/science.1098410 15591194

[B9] Espy K. A. (1997). The shape school: assessing executive function in preschool children. *Dev. Neuropsy.* 13 495–499 10.1080/87565649709540690

[B10] Flavell J. H. (2000). Development of children’s knowledge about the mental world. *Int. J. Behav. Dev.* 24 15–23 10.1080/016502500383421

[B11] Flavell J. H. Everett B. A. Croft K. Flavell E. R. (1981). Young childrens knowledge about visual-perception – further evidence for the level 1-level 2 distinction. *Dev. Psychol.* 17 99–103 10.1037/0012-1649.17.1.99

[B12] Flavell J. H. Flavell E. R. Green F. L. (1983). Development of the appearance reality distinction. *Cogn. Psychol.* 15 95–120 10.1016/0010-0285(83)90005-1 6831859

[B13] Hamilton A. F. D. Brindley R. Frith U. (2009). Visual perspective-taking impairment in children with autistic spectrum disorder. *Cognition* 113 37–44 10.1016/j.cognition.2009.07.007 19682673

[B14] Hands L. J. (1972). Discrimination of left and right and the development of the logic of relations. *Merrill Palmer Q.* 18 307–320

[B15] Hare B. Tomasello M. (2005). Human-like social skills in dogs? *Trends Cogn. Sci.* 9 439–444 10.1016/j.tics.2005.07.003 16061417

[B16] Harris L. J. Strommen E. A. (1972). Role of front-back features in childrens front, back, and beside placements of objects. *Merrill Palmer Q. Behav. Dev.* 18 259–271

[B17] Huttenlocher J. Presson C. C. (1973). Mental rotation and perspective problem. *Cogn. Psychol.* 4 277–299 10.1016/0010-0285(73)90015-7

[B18] Kaminski J. Riedel J. Call J. Tomasello M. (2001). Domestic goats (Capra hircus) follow gaze direction and use some social cues in an object choice task. *Anim. Behav.* 69 11–18 10.1016/j.anbehav.2004.05.008

[B19] Keehner M. Guerin S. Miller M. Turk D. Hegarty M. (2006). Modulation of neural activity by angle of rotation during imagined spatial transformations. *NeuroImage* 33 391–398 10.1016/j.neuroimage.2006.06.043 16935007

[B20] Kessler K. Apperly I. A. Butterfill S. A. (under review) Cross-disciplinary views on visual perspective-taking: where and how to look for the embodied origins of mentalizing.

[B21] Kessler K. Rutherford H. (2010). The two forms of visuo-spatial perspective-taking are differently embodied and subserve different spatial prepositions. *Front. Psychol.* 1 1–12 21833268 10.3389/fpsyg.2010.00213 PMC3153818

[B22] Kessler K. Thomson L. A. (2010). The embodied nature of spatial perspective-taking: embodied transformation versus sensorimotor interference. *Cognition* 114 72–88 10.1016/j.cognition.2009.08.015 19782971

[B23] Kessler K. Wang H. (2012). Spatial perspective-taking is an embodied process, but not for everyone in the same way: differences for gender and social skills score. *Spat. Cogn. Comput.* 12 133–158 10.1080/13875868.2011.634533

[B24] Kirkham N. Z. Cruess L Diamond A. (2003). Helping children apply their knowledge to their behavior on a dimension-switching task. *Dev. Sci.* 6 449–467 10.1111/1467-7687.00300

[B25] Kozhevnikov M. Hegarty M. (2001). A dissociation between object manipulation spatial ability and spatial orientation ability. *Mem. Cognit.* 29 745–756 10.3758/BF03200477 11531229

[B26] Levinson S. C. (1996). Language and space. *Annu. Rev. Anthropol.* 25 353–382 10.1146/annurev.anthro.25.1.353

[B27] Levinson S. C. (2003). *Space in Language and Cognition: Explorations in Cognitive Diversity*. Cambtridge: Cambridge University Press 10.1017/CBO9780511613609

[B28] Light P. Nix C. (1983). Own view versus good view in a perspective-taking task. *Child Dev.* 54 480–483 10.2307/1129709 6872635

[B29] Masangkay Z. S. Mccluske K. A. Mcintyre C. W. Simsknig J. Vaughn B. E. Flavell J. H. (1974). Early development of inferences about visual percepts of others. *Child Dev.* 45 357–366 10.2307/1127956 4837714

[B30] Michelon P. Zacks J. M. (2006). Two kinds of visual perspective-taking. *Percept. Psychophys.* 68 327–337 10.3758/BF03193680 16773904

[B31] Moll H. Meltzoff A. N. (2011). How does it look? Level 2 perspective-taking at 36 months of age. *Child Dev.* 82 661–673 10.1111/j.1467-8624.2010.01571.x 21410927

[B32] Moll H. Tomasello M. (2006). Level I perspective-taking at 24 months of age. *Br. J. Dev. Psychol.* 24 603–613 10.1348/026151005X55370

[B33] Perner J. (1991). *Understanding the Representational Mind*. Cambridge, MA: MIT Press

[B34] Perrucci V. Agnoli F. Albiero P. (2008). Children’s performance in mental rotation tasks: orientation-free features flatten the slope. *Dev. Sci.* 11 732–742 10.1111/j.1467-7687.2008.00723.x 18801129

[B35] Piaget J. Inhelder B. (1956). *The Child’s Conception of Space*. London: Routledge & Kegan Paul

[B36] Premack D. Woodruff G. (1978). Does the chimpanzee have a theory of mind. *Behav. Brain Sci.* 1 515–526 10.1017/S0140525X00076512

[B37] Riggs K. J. Peterson D. M. Robinson E. J. Mitchell P. (1998). Are errors in false belief tasks symptomatic of a broader difficulty with counterfactuality? *Cogn.Dev.* 13 73–91 10.1016/S0885-2014(98)90021-1

[B38] Russell J. (1996). *Agency: Its Role in Mental Development*. Hove: Erlbaum

[B39] Samson D. Apperly I. A. Braithwaite J. J. Andrews B. J Scott S. E. B. (2010). Seeing it their way: evidence for rapid and involuntary computation of what other people see. *J. Exp. Psychol. Hum. Percept. Perform.* 36 1255–1266 10.1037/a0018729 20731512

[B40] Simon J. R. (1969). Reactions towards the source of stimulation. *J. Exp. Psychol.* 81 174–176 10.1037/h0027448 5812172

[B41] Song H. J. Baillargeon R. (2008). Infants’ reasoning about others’ false perceptions. *Dev. Psychol.* 44 1789–1795 10.1037/a0013774 18999340 PMC3372913

[B42] Surtees A. D. R. Apperly I. A. (2012). Egocentrism and automatic perspective-taking in children and adults. *Child Dev.* 83 452–460 10.1111/j.1467-8624.2011.01730.x. 22335247

[B43] Surtees A. D. R. Apperly I. A. Samson D. (2013). Similarities and differences in visual and spatial perspective-taking processes. *Cognition* 129 426–438 10.1016/j.cognition.2013.06.008 23999408

[B44] Surtees A. D. R. Butterfill S. A. Apperly I. A. (2012a). Direct and indirect measures of Level-2 perspective-taking in children and adults. *Br. J. Dev. Psychol.* 30 75–86 10.1111/j.2044-835X.2011.02063.x 22429034

[B45] Surtees A. D. R. Noordzij M. L. Apperly I. A. (2012b). How to lose yourself in space: children and adults’ use of frames of reference and perspectives. *Dev. Psychol.* 48, 185 191 10.1037/a0025863 22004339

[B46] Tomasello M. (2008). *Origins of Human Communication*. Cambridge: MIT Press

[B47] Tomasello M. Call J. Hare B. (2003). Chimpanzees understand psychological states – the question is which ones and to what extent. *Trends Cogn. Sci.* 7 153–156 10.1016/S1364-6613(03)00035-4 12691762

[B48] Tversky B. Hard B. M. (2009). Embodied and disembodied cognition: spatial perspective-taking. *Cognition* 110 124–129 10.1016/j.cognition.2008.10.008 19056081

[B49] Weisberg D. P. Beck S. R. (2010). Children’s thinking about their own and others’ regret and relief. *J. Exp. Child Psychol.* 106 184–191 10.1016/j.jecp.2010.02.005 20307891

[B50] Wimmer H. Perner J. (1983). Beliefs about beliefs – representation and constraining function of wrong beliefs in young childrens understanding of deception. *Cognition* 13 103–128 10.1016/0010-0277(83)90004-5 6681741

[B51] Zacks J. Mires J. Tversky B. Hazeltine E. (2000). Mental spatial transformations of objects and perspective. *Spat. Cogn. Comput.* 2 315–332 10.1023/A:1015584100204

[B52] Zacks J. M. Michelon P. (2005). Transformations of visuospatial images. *Behav. Cogn. Neurosci. Rev.* 4 96–118 10.1177/1534582305281085 16251727

